# Spectral-domain optical coherence tomography evaluation of postoperative cystoid macular oedema following phacoemulsification with intraoperative complication

**DOI:** 10.1186/1471-2415-14-16

**Published:** 2014-02-17

**Authors:** Keat Ween Khaw, Hee Hong Lam, Tsung Fei Khang, Azida Juana Wan Ab Kadir, Visvaraja Subrayan

**Affiliations:** 1 Department of Ophthalmology, University of Malaya Medical Centre, Lembah Pantai, Kuala Lumpur 59100, Malaysia; 2 Institute of Mathematical Sciences, Faculty of Science, University of Malaya, Kuala Lumpur, Malaysia

**Keywords:** Cystoid macular oedema, Spectral-domain optical coherence tomography, Phacoemulsification, Intraoperative complication

## Abstract

**Background:**

To report the rate of cystoid macular oedema (CMO) as detected by spectral-domain optical coherence tomography (SD-OCT) after intraoperative complication during phacoemulsification. The secondary objectives include comparing mean macular thickness and best-corrected visual acuity (BCVA) between those who developed postoperative CMO against those who did not.

**Methods:**

This is a prospective cohort study conducted in a tertiary hospital between July 2009 and June 2010. Serial SD-OCT and BCVA were performed at baseline, 1 week, 6 weeks and 16 weeks postoperatively.

**Results:**

Single eyes from 47 subjects were analyzed; of these 16 (34%) eyes developed CMO. In the CMO group, mean macular thickness (±SD) increased sharply by 56 μm from 273 ± 24 μm at baseline to 329 ± 31 μm at 16 weeks; whereas in the non-CMO group, macular thickness showed a slight increase of 14 μm from 259 ± 21 μm to 272 ± 20 μm. In the CMO group, mean BCVA (in logarithm of minimum angle of resolution) improved modestly from 0.92 ± 0.66 to 0.66 ± 0.41 at week 16; while in the non-CMO group, mean BCVA improved markedly from 0.98 ± 0.59 to 0.21 ± 0.13. The two groups differed significantly in mean macular thickness (p < 0.001) and mean BCVA (p < 0.001) at 16 weeks.

**Conclusion:**

As detection rate of CMO is high, postoperative OCT monitoring for patients with intraoperative complications allows earlier diagnosis and treatment.

## Background

Postoperative cystoid macular oedema (CMO) is an important cause of disappointing vision after phacoemulsification, especially if intraoperative complication occurs. The incidence of CMO varies widely across literature. This in part is due to the various ways of defining and diagnosing CMO as well as the type of cataract surgery performed [1]. Clinical and clinically-significant CMO are terms used for patients with angiographic evidence of CMO accompanied by a decrease in best-corrected visual acuity (BCVA) [2]. For cataract surgery, this is usually taken as being worse than logMAR of 0.3 (or Snellen visual acuity of 20/40) [3].

Traditionally, fluorescein angiography (FA) is used to confirm the presence of perifoveal leakage. In severe cases, this is classically seen as a petaloid pattern of hyperfluorescence in the late frames. Currently, optical coherence tomography (OCT) is a non-invasive method that can give high-resolution, cross-sectional images of the retina and its thickness. Spectral-domain OCT (SD-OCT) provides an axial resolution of 5 μm, thus allowing better visualization of the retinal layers to specifically detect subtle intraretinal changes [4]. It is superior to FA in the diagnosis of CMO in that it is easy to perform, safe, objective and quantifiable. Furthermore, Jittpoonkuson *et al.* reported that OCT is more sensitive than FA in detecting CMO associated with retinal vein occlusion, age-related macular degeneration and diabetic retinopathy. They found that CMO was missed by FA in 18.5%, 33.33% and 33.33% of these cases respectively [5].

Using OCT, transient and clinically insignificant changes in the macular thickness have been observed after uneventful phacoemulsification [6,7]. In a series by Ching *et al.*, only 3% of cases (4/131) demonstrated tomographically-evident-CMO by 8 weeks after uncomplicated phacoemulsification [8]. Following vitreous loss, the incidence of clinical CMO is higher, between 6-21% [9]. However, these studies lack uniformity in terms of the type of cataract surgery performed (phacoemulsification, extracapsular cataract surgery or intracapsular cataract surgery) as well as how CMO was diagnosed (clinical, angiographic or OCT).

We are unable to find any prospective study that utilizes OCT to detect the rate of CMO after intraoperative complication during phacoemulsification. Hence our study aims to address this issue. Our secondary objectives are to compare the mean macular thickness and BCVA of patients who developed postoperative CMO against those who did not.

## Methods

We conducted a prospective cohort study which adhered to the Declaration of Helsinki. Institutional Review Board approval was obtained and all subjects gave informed consent prior to enrollment into the study.

Study participants consisted of consecutive cohort of patients who underwent phacoemulsification with intraoperative complication in the University of Malaya Medical Center between July 2009 and June 2010. Intraoperative complication was defined based on similar studies and included the presence of any of the following conditions, whether in isolation or in combination: posterior capsule rent, zonulodialysis, presence of vitreous loss, dropped nucleus, complications needing enlargement of the corneal wound or a second operation and position of the intraocular lens other than in-the-bag [10,11].

All patients 21 years of age or older with sufficient media clarity to permit preoperative OCT evaluation were eligible for inclusion in the study. Our exclusion criteria included patients on prostaglandin analogues, topical or systemic steroid or non streroidal anti-inflammatory drugs, history of uveitis, prior intraocular injections or surgery, past or pre-existing retinal and choroidal diseases that could affect retinal thickness. These included but are not limited to CMO, diabetic retinopathy or maculopathy, retinal vein occlusion, age-related macular degeneration, radiation retinopathy, posterior uveitis and previous laser treatment. Subjects with abnormal macula based on clinical examination and preoperative macular OCT scans were excluded from this study.

Initial screening was conducted during subjects’ preoperative visits two weeks prior to their scheduled phacoemulsification. We obtained patient’s detailed history and performed ophthalmologic examination that included BCVA, slit-lamp biomicroscopy, intraocular pressure measurement, dilated fundus examination and OCT scans. Throughout this study, BCVA was performed with refraction. It was measured using a back-illuminated Snellen chart at 6 meters and converted to logarithm of minimal angle of resolution (logMAR) scale for analysis. SD-OCT (Cirrus; Carl Zeiss Meditech, Inc, Jena, Germany) scanning was done using the macular cube scan (512×128 scan pattern) centered on the fovea. Scans with signal strength 6 or greater were deemed acceptable. All OCT scans for this study were performed by two of the authors (KWK and HHL).

From the preoperative screening, 805 subjects who met the inclusion and exclusion criteria were identified. All consented for the study. From these, 48 eyes of 48 patients experienced intraoperative complication during phacoemulsifiction. They were subsequently reviewed at 1 week, 6 weeks and 16 weeks postoperatively [6-8]. At each visit, BCVA, slit-lamp examination and OCT scan were performed. One patient did not complete the scheduled follow up. A patient is defined as belonging to the CMO group if he or she developed CMO at week 16.

After phacoemulsification, all patients received standardized topical steroid and antibiotic drops. This involved two-hourly topical administration of prednisolone acetate 1% (Pred Forte, Allergan, Irvine, CA) for the first week, four times daily for one month and then subsequently tapered. Topical antibiotics were given four times daily during the same period.

Upon the detection of CMO, a step-wise treatment approach was employed. In the first instance, correctable causes like vitreous traction at the wound were addressed. Subsequently, topical steroid application was intensified and topical non-steroidal anti-inflammatory drug was added. No patients with CMO in our series needed more invasive approaches such as the use of sub-Tenon or intravitreal triamcinolone and/or intravitreal anti-vascular endothelial growth factor.

Data collection included demographic characteristics, ocular and medical history as well as intraoperative details such as surgeon’s experience (resident or consultant ophthalmologist), operation time, type of complication and intraocular lens position.

The main outcome measure was the rate of postoperative CMO. This was defined as the presence of hyporeflective intraretinal cystoid changes noted on SD-OCT which was not present preoperatively [12]. It was graded by a single consultant ophthalmologist (VS) who was masked from the patient’s intraoperative complication, visual acuities and treatment approach. Secondary outcomes included mean macular thickness and BCVA, compared between patients who developed postoperative CMO against those who did not.

Since the patterns of visual improvement dependency on change in macular thickness for the CMO and non-CMO group across four time points may be complex, we explored the data graphically using the scatter plot and the heat plot. The latter is a powerful graphical method that is commonly used for exploring patterns in multidimensional data, such as those from genomic studies [13]. For statistical analysis, paired t-test was used to test for significant difference in mean macular thickness and BCVA between baseline and at 16 weeks for both the CMO and non-CMO groups. To compare the discrepancy of mean macular thickness between these two groups at baseline and at 16 weeks, we used the two-sample t-test with Bonferonni adjustment (5% significance level). All graphical plots and statistical analyses were done using R Version 2.13.2 [14].

## Results

During the one-year enrollment period, 805 patients who met the eligibility criteria were identified. All consented for the study. From these, a total of 48 eyes of 48 patients encountered intraoperative complication during phacoemulsification. One patient transferred her follow up to another hospital due to logistical reason. Consequently, 47 eyes of 47 patients completed their 1-week, 6-weeks and 16-weeks follow up. One patient missed his 6-week follow up visit. The mean age of patients was 67 ± 8 years (range: 45 to 82 years) and 49% of them were males.

Of the 48 eyes that underwent phacoemulsification, 25 (47%) were performed on the left eye. Residents operated on 31 (66%) eyes, while consultant ophthalmologists operated on 16 eyes. The distribution of operation time (±SD) for phacoemulsification was bimodal (80 mins and below: n = 34, 55 ± 14 mins; above 80 mins: n = 13, 123 ± 18 mins). The observed intraoperative complications are as follows: posterior capsule rent (n = 40), zonulodialysis (n = 8), vitreous loss (n = 35), enlargement of corneal wound (n = 35) and dropped nucleus (n = 7). Vitreous loss accompanied 82% of posterior capsule rent and 88% of zonulodialysis. With regards to the size of nucleus drop, three cases were larger than a quarter nucleus size.

There were seven cases that needed a second surgery. The reasons for surgery included dropped nucleus (n = 3), retained cortex (n = 2), wound leak (n = 1) and secondary lens implantation (n = 1). All were performed within a week from the first surgery. With regards to intraocular lens implantation, 9 cases had in-the-bag implantation, 24 in the sulcus and 14 in the anterior chamber. No eyes were left aphakic. None of the subjects developed post-operative complications such as endophthalmitis or corneal decompensation.

**Figure 1:**
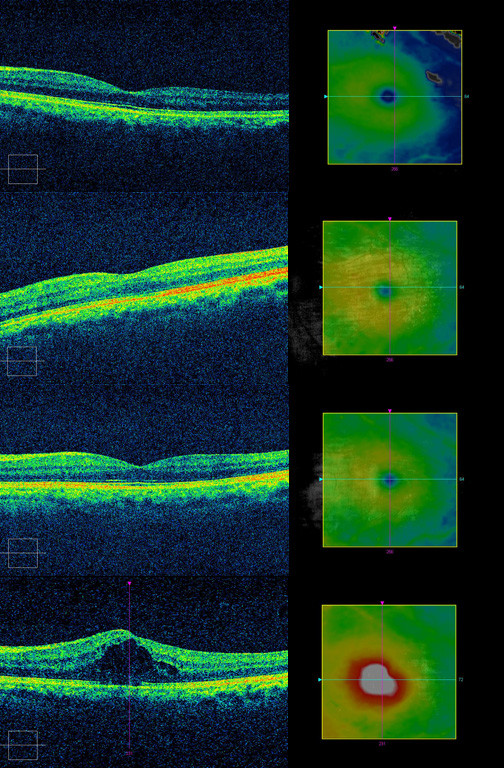
Serial macular OCT scans at baseline, postoperative 1 week, 6 weeks and 16 weeks for patient #35 with postoperative CMO.

Acceptable OCT scans were available for all eyes at all time intervals except 6 eyes at 1-week follow up due to hazy corneas. In total, 16 eyes (34.0%) developed postoperative CMO. Cystoid spaces were detected on SD-OCT in two eyes at week 1, nine eyes at week 6 and five eyes at week 16. One eye demonstrated resolution of CMO by week 16. An example of the serial OCT scans of a subject who developed postoperative CMO is shown in Figure 1.

At baseline, mean macular thickness in the CMO group was 273 ± 24 μm; in the non-CMO group, it was 259 ± 21 μm. There was no significant difference in mean macular thickness between both groups (p = 0.06). Following complicated phacoemulsification, mean macular thickness at 16 weeks in the CMO group increased 55 μm, to 329 ± 31 μm; this increase was biologically significant (95% CI: [38,73]; p < 0.001). In the non-CMO-group, the increase was only 14 μm, to 272 ± 20 μm; this increase was statistically significant (p = 0.01), but relatively unimportant from a biological point of view (95% CI:[3,24]). Mean macular thickness in the CMO group was significantly thicker than the non-CMO group by 56 μm (95% CI: [36, 77]; p < 0.001).

Similarly, baseline BCVA in the CMO group was 0.92 ± 0.66; in the non-CMO group, it was 0.98 ± 0.59. There was no significant difference in BCVA between both groups (p = 0.7). Following complicated phacoemulsification, mean BCVA at 16 weeks in the CMO group decreased to 0.66 ± 0.41. However, this improvement was not statistically significant (p = 0.2). In the non-CMO group, mean BCVA decreased substantially to 0.21 ± 0.13. The magnitude of decrease was 0.78, a result that was both statistically and clinically significant (95% CI: [-0.98,-0.21]; p < 0.001). Mean BCVA in the non-CMO group was significantly less by 0.45 compared to the CMO group (95% CI: [-0.71,-0.19]; p < 0.001) by 16 weeks postoperatively. Furthermore, about 94% of non-CMO patients ended with BCVA of 0.3 or better, compared to only 38% in the CMO group.

**Figure 2:**
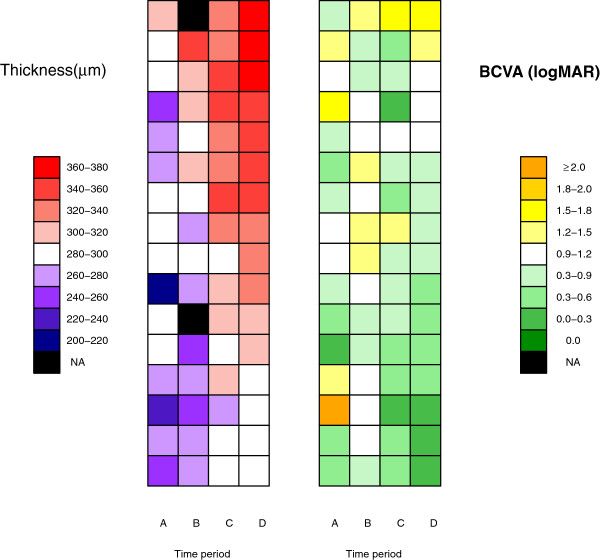
**Heat map of macular thickness and BCVA profiles for CMO patients.** Symbols: A for baseline; B for Week 1; C for Week 6; D for Week 16; NA for missing data due to hazy cornea or appointment default. Each row corresponds to a subject ranked in descending order according to macular thickness at week 16.

**Figure 3:**
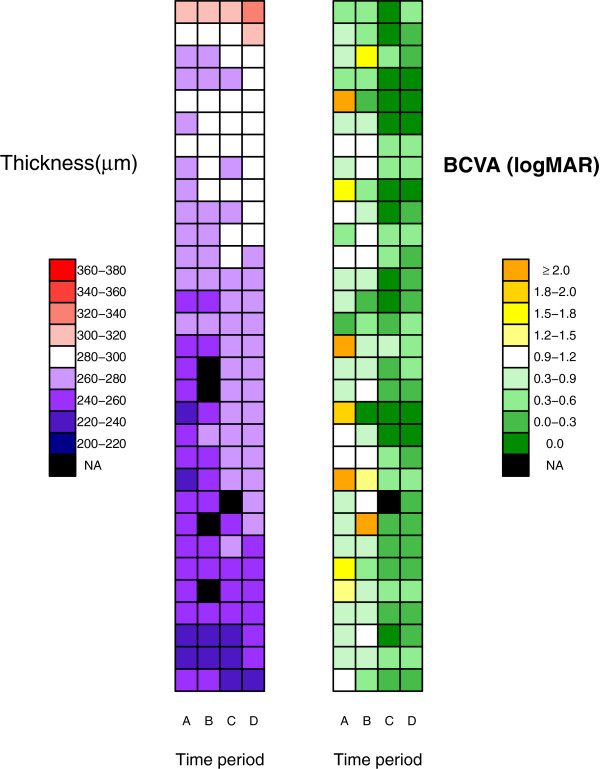
**Heat map of macular thickness and BCVA profiles for non-CMO patients.** Symbols: A for baseline; B for Week 1; C for Week 6; D for Week 16; NA for missing data due to hazy cornea or appointment default. Each row corresponds to a subject ranked in descending order according to macular thickness at week 16.

Figures 2 and 3 show the macular thickness and BCVA profiles of patients in the CMO and non-CMO groups, respectively. The patients are ranked in descending order according to macular thickness at week 16. Comparing both figures, we note that macular thickening is generally more pronounced in CMO patients compared to non-CMO patients, as indicated by multiple transitions of colour tones across the four time periods. Similarly, BCVA improvement appears to be concentrated in CMO patients who had more gradual rather than abrupt thickening of the macular.

## Discussion

### Incidence of CMO after complicated phacoemulsification

This prospective cohort study is, to our knowledge, the first to report on the incidence of CMO as detected by OCT and its serial macular thickness in patients with intraoperative complications during phacoemulsification. In our study the rate of tomographic CMO was about 31%. In the literature, the reported percentage of clinical CMO following intraoperative complication varies widely from 1.7% to 10% [2,9,15-18]. We note that these studies were designed primarily to study visual outcome and not specifically to detect CMO. Also, they are retrospective in nature, and did not provide details as to how CMO was diagnosed. Another drawback from studies such as those by Chan *et al*. [17] and Blomquist and Rugwani [9] is the fact that they included various forms of cataract surgery like extracapsular and intracapsular cataract extractions, which carry higher risk of CMO.

We believe our rate of detecting CMO was much higher as a consequence of using SD-OCT. Typically, percentages of angiographic and tomographic CMO are much higher than clinical CMO. In one study using FA, the rate of angiographic CMO at 6 weeks after uncomplicated cataract surgery was 18.7%, although the rate of clinical CMO was only 2.1% [3]. More specifically, in the subgroup with intraoperative complications, the rate of angiographic CMO and clinical CMO was 11% and 4.8%, respectively. Previously, Ching *et al.* used OCT to detect CMO following uneventful phacoemulsification [8]. They found 4 out of 131 patients (3%) developed CMO. Unfortunately, these patients were excluded from the analysis and the serial macular thickness was not given. Minnella *et al.* gave OCT details of 15 patients with Irvine-Gass syndrome [12]. However they did not mention whether these patients had intraoperative complication during surgery.

### Evaluation of serial macular thickness

Besides identifying CMO, OCT enables quantitative evaluation of macular thickness over time, a feature not possible with FA. For patients who did not develop CMO in our cohort, their serial mean macular thickness showed a small increase over time (259 μm to 272 μm in 4 months). This mean change of 14 μm, although statistically significant, is not clinically significant and is comparable with other published studies looking at serial changes of macular thickness following uneventful phacoemulsification [6-8].

In the CMO group, we found a steady increase in mean macular thickness over time. It is often quoted that postoperative CMO only develops 6–8 weeks after surgery [19]. However in our series we found the onset of CMO to be gradual rather than occurring at a specific point. This concurs with newer studies using OCT that detected a greater percentage of patients with CMO occurring at an earlier postoperative period. Ching *et al.* found that CMO can develop as early as the second postoperative week [8].

The visualization of patients serial data using the heat map allows the possibility of correlating patterns of change in macular thickness with other clinical symptoms of interest in the future. Such analyses are missed and not possible with routine statistical tests.

### Visual acuity outcome after complicated phacoemulsification

In our study, the overall proportion of patients who achieved BCVA of 0.3 or better was 74% (35/47). In cases where CMO developed, their BCVA was expectedly worse. However, in our subgroup that did not develop CMO, the percentage of good visual outcome is comparable to other published data (6% did not achieve BCVA of 0.3 or less). Tan and Karwatowski reported a study from the United Kingdom that showed that 14% of their 92 patients with complicated phacoemulsification requiring anterior vitrectomy did not achieve BCVA of 0.3 or less [18]. Moreover, of those who developed pseudophakic CMO, all had VA between 0.5 to 0.8. Another study from Singapore showed that 13% of 115 patients with posterior capsule rupture failed to achieve BCVA of 0.3 or less [17]. In a large series from Sweden involving 171 patients with capsule complication, 27% did not achieve BCVA of 0.5 or less [20].

### Limitations

Our study has several limitations. Firstly, in order to have larger sample size, we were unable to conduct a single surgeon study. As such, variability in how complications were handled and the differences in postoperative management were to be expected. Secondly, we did not follow the remaining 753 subjects who consented for the study but did not encounter any intraoperative complication during their surgery. Previously our centre reported serial macular thickness measurements using SD-OCT in 132 patients who underwent uneventful phacoemulsification [21]. No CMO cases were detected in this series. As such we felt that it was a burden to resource and unethical to subject a large group of cohort to repeated OCT testing.

## Conclusion

Nonetheless, we believe the results from this study are potentially valuable to the surgeon as it highlights the need to be vigilant in detecting postoperative CMO in patients whounderwent complicated phacoemulsification. We recommend routine postoperative OCT monitoring in these patients as the detection rate of CMO is high and associated with a corresponding decrease in visual acuity. The prompt diagnosis would allow earlier treatment to be instituted.

## Competing interest

The authors declare that they have no competing interest.

## Authors’ contribution

KWK conceived the study, collected and analyzed the data and wrote the manuscript. HHL participated in the study design, collected the data and contributed to the manuscript draft. TFK analyzed the data and edited the manuscript. WAKAJ participated in the study design and edited the manuscript. VS participated in the study design, analyzed the OCT scans and edited the manuscript. All authors read and approved the final manuscript.

## Pre-publication history

The pre-publication history for this paper can be accessed here:

http://www.biomedcentral.com/1471-2415/14/16/prepub

## References

[B1] Rossetti L Autelitano A Cystoid macular edema following cataract surgery Curr Opin Ophthalmol 2000 11 65 72 10.1097/00055735-200002000-00010 10724830

[B2] Henderson BA Kim JY Ament CS Ferrufino-Ponce ZK Grabowska A Cremers SL Clinical pseudophakic cystoid macular edema: risk factors for development and duration after treatment J Cataract Refract Surg 2007 33 1550 1558 10.1016/j.jcrs.2007.05.013 17720069

[B3] Wright PL Wilkinson CP Balyeat HD Popham J Reinke M Angiographic cystoid macular edema after posterior chamber lens implantation Arch Ophthalmol 1988 106 740 744 10.1001/archopht.1988.01060130810028 3369996

[B4] Gabriele ML Wollstein G Ishikawa H Xu J Kim J Kagemann L Three dimensional optical coherence tomography imaging: advantages and advances Prog Retin Eye Res 2010 29 556 57 10.1016/j.preteyeres.2010.05.005 20542136 PMC2962728

[B5] Jittpoonkuson T Garcia PMT Rosen RB Correlation between fluorescein angiography and spectral-domian optical coherence tomography in the diagnosis of cystoid macular edema Br J Ophthalmol 2010 94 1197 1200 10.1136/bjo.2009.170589 19965832

[B6] Biro Z Balla Z Kovacs B Change of foveal and perifoveal thickness measured by OCT after phacoemulsification and IOL implantation Eye 2008 22 8 12 10.1038/sj.eye.6702460 16751754

[B7] von Jagow B Ohrloff C Kohnen T Macular thickness after uneventful cataract surgery determined by optical coherence tomography Graefes Arch Clin Exp Ophthalmol 2007 245 1765 1771 10.1007/s00417-007-0605-6 17619896

[B8] Ching H-Y Wong AC Wong C-C Woo DC Chan CW Cystoid macular oedema and changes in retinal thickness after phacoemulsification with optical coherence tomography Eye 2006 20 297 303 10.1038/sj.eye.6701864 15818389

[B9] Blomquist PH Rugwani RM Visual outcomes after vitreous loss during cataract surgery performed by residents J Cataract Refract Surg 2002 28 847 852 10.1016/S0886-3350(01)01117-8 11978467

[B10] Rutar T Porco TC Naseri A Risk factors for intraoperative complications in resident-performed phacoemulsification surgery Ophthalmol 2009 116 431 436 10.1016/j.ophtha.2008.10.028 19167084

[B11] Woodfield AS Gower EW Cassard SD Ramanthan S Intraoperative phacoemulsification complication rates of second- and third-year ophthalmology residents: a 5-year comparison Ophthalmol 2011 118 954 958 10.1016/j.ophtha.2010.08.047 21539981

[B12] Minnella AM Savastano MC Zinzanella G Mazzone G Federici M Gari M Spectral-Domain optical coherence tomography in Irvine–Gass Syndrome Retina 2012 32 581 587 10.1097/IAE.0b013e31821e2225 21963486

[B13] Schroeder MP Gonzalez-Perez A Lopez-Bigas N Visualizing multidimensional cancer genomics data Genome Med 2013 5 9 10.1186/gm413 23363777 PMC3706894

[B14] R Core Team R: A Language and Environment for Statistical Computing 2012 Vienna, Austria: R Foundation for Statistical Computing http://www.r-project.org

[B15] Konstantopoulos A Yadegarfar G Madhusudhana K Canning CR Luff AJ Anderson DF Prognostic factors that determine visual outcome following cataract surgery complicated by vitreous loss Eur J Ophthalmol 2009 19 2 247 25 19253242 10.1177/112067210901900212

[B16] Onal S Gozum N Gucukoglu A Visual results and complications of posterior chamber intraocular lens implantation after capsular tear during phacoemulsification Ophthalmic Surg Lasers Imaging 2004 35 3 219 24 15185790

[B17] Chan FM Mathur R Ku JJK Chen C Chan SP Yong VSH Short-term outcomes in eyes with posterior capsule rupture during cataract surgery J Cataract Refract Surg 2003 29 537 41 10.1016/S0886-3350(02)01622-X 12663021

[B18] Tan JHY Karwatowski WSS Phacoemulsification cataract surgery and unplanned anterior vitrectomy: is it bad news? Eye 2002 16 117 20 10.1038/sj/eye/6700015 11988808

[B19] Klein RM Yannuzzi L Cystoid macular edema in the first week after cataract extraction Am J Ophthalmol 1976 81 614 5 1275040 10.1016/0002-9394(76)90126-4

[B20] Johansson B Lundström M Montan P Stenevi U Behndig A Capsule complication during cataract surgery: long-term outcomes: Swedish Capsule Rupture Study Group report 3 J Cataract Refract Surg 2009 35 1694 1698 10.1016/j.jcrs.2009.05.027 19781461

[B21] Lam HH Khaw GKW Khang TF Subrayan V Evaluation of macular thickness by SD-OCT Ophthalmology 2012 119 1 197 198 22214942 10.1016/j.ophtha.2011.09.003

